# Cannabis and Dermatological Implications: A Traditional Review of Adverse Cutaneous Reactions and Systemic Risks

**DOI:** 10.7759/cureus.82711

**Published:** 2025-04-21

**Authors:** Shitij Shrivastava, Shashwat Shrivastava, Monika Shrestha, Satyam Mahaju

**Affiliations:** 1 Internal Medicine, BronxCare Health System, New York City, USA; 2 Medicine, California Institute of Behavioral Neurosciences and Psychology, Fairfield, USA; 3 Cardiothoracic Surgery, New York University (NYU) Langone Health, New York City, USA

**Keywords:** cannabis research, dermatology, marijuana use, skin, tetrahydrocannabinol (thc)

## Abstract

The growing use of cannabis for both recreational and medicinal purposes has raised concerns about its cutaneous adverse effects. While systemic implications of cannabis are widely reported, dermatological manifestations remain underrecognized and underreported. This review aims to consolidate existing literature on cannabis-related dermatological complications, including allergic contact dermatitis, urticaria, Stevens-Johnson syndrome (SJS), and cannabis-induced arteritis. Emphasis is placed on mechanisms involving the endocannabinoid system in the skin, case reports of dermatological reactions, and implications for clinical dermatology. A structured methodology was applied to source peer-reviewed articles from databases including PubMed and Scopus, selecting studies published between 2000 and 2024 that directly addressed dermatological outcomes of cannabis use. Findings suggest a growing need for clinician awareness of cannabis as a potential etiologic agent in dermatological pathology. Future dermatology-focused research is essential to understand causality, diagnostic criteria, and effective treatment strategies.

## Introduction and background

As research in dermatology continues to evolve, intriguing connections between the skin and various compounds found in nature are coming to light. Among these compounds, cannabinoids, particularly those found in marijuana (*Cannabis sativa*), have been of significant interest among dermatologists and healthcare personnel. Cannabis is a complex plant with over a hundred identified cannabinoids, such as delta-9-tetrahydrocannabinol (THC) and cannabidiol (CBD), each with distinct physiological effects. THC is responsible for marijuana’s psychoactive properties. CBD is used for its non-psychoactive, therapeutic potential. Recent advances in research have unveiled a diverse range of interactions between cannabinoids and the skin [1]. Cannabinoid receptors, notably cannabinoid receptor 1 (CB1) and cannabinoid receptor 2 (CB2), part of the endocannabinoid system, are distributed throughout the skin’s various cell types, including keratinocytes, sebocytes, and immune cells, and are the site where cannabis acts [2]. Cannabis is associated with arterial disease, including thromboangiitis obliterans and atheromatosis, causing cutaneous manifestations, most commonly seen in teenagers [3]. A suggested mechanism involves the influence of CB1 and CB2 on the platelet expression of glycoprotein IIb-IIIa and P-selectin. When combined with endothelial damage and heightened heart rate and sympathomimetic activity, this interaction could potentially trigger myocardial and peripheral vascular events. It can also cause immunoglobulin E-mediated allergic reactions [4].

Objective

This study aims to review and synthesize the dermatological complications associated with cannabis use, drawing from case reports, clinical studies, and basic science research.

Research question

What are the dermatological complications associated with cannabis use, and how are these manifestations supported by clinical, mechanistic, and epidemiological evidence?

## Review

Methodology 

We conducted a narrative review following the Preferred Reporting Items for Systematic Reviews and Meta-Analyses extension for Scoping Reviews (PRISMA-ScR) guidelines. A comprehensive literature search was performed using PubMed, Embase, Scopus, and Google Scholar databases. Keywords included “cannabis,” “marijuana,” “THC,” “CBD,” “skin,” “dermatology,” “cutaneous,” “urticaria,” “Stevens-Johnson Syndrome,” and “contact dermatitis.” Articles published between January 2000 and March 2024 were included. Studies were selected based on relevance to dermatological outcomes.

Review

Several cases [5] have described various forms of skin rashes, including erythematous and pruritic skin lesions, papules, morbilliform exanthem, and monomorphic urticarial lesions, associated with the use of cannabis, especially with pharmaceutical-grade CBD. These manifested on the trunk and abdomen, with an outward progression toward the limbs while sparing the face and palmoplantar regions. These areas also exhibited either pruritus or heightened sensitivity to touch [5]. In one study, authors have reported cannabis use to be associated with fungal infections such as aspergillosis, mucormycosis, histoplasmosis, cryptococcosis, coccidioidomycosis, and blastomycosis. It is hard to associate causality between cannabis use and fungal infections. According to this study, the comparable geographical prevalence of fungal infections in individuals using cannabis and those who abstain from cannabis is noteworthy [6]. It becomes crucial for healthcare providers to comprehend the comprehensive range of potential advantages and drawbacks linked to cannabis use, including the risk of abuse [7]. Clinical studies have demonstrated promising results in the role of cannabis for the treatment of psoriasis, atopic dermatitis, and acne [8]. According to the American Academy of Allergy, Asthma, and Immunology, marijuana allergy is associated with immunoglobulin E-specific antibodies. Symptoms may include rhinorrhea, epiphora, and itching. However, life-threatening reactions are limited to hempseed in marijuana allergic individuals [9].

Cannabis, one of humanity’s oldest cultivated plants, has a rich and varied history dating back thousands of years. Its use spans diverse cultures and civilizations, with evidence of cannabis cultivation and consumption found in ancient China, India, and Egypt [10-12]. The plant’s journey continued across continents, reaching Europe and eventually the Americas through colonial trade routes. In the United States, cannabis was initially cultivated for industrial purposes, with its fibers used in rope, textiles, and paper production. However, its medicinal properties were also recognized, leading to its inclusion in various pharmaceutical preparations during the 19th century. In recent decades, attitudes toward cannabis have evolved, with growing recognition of its therapeutic potential and calls for legalization and regulation [11]. The history of cannabis is a complex tapestry of cultural, social, and political influences spanning millennia. Inhalation, the primary method of administering cannabis, offers a swift and effective means of delivering the drug from the lungs to the brain, thereby increasing its potential for misuse. This rapid transfer of the drug to the central nervous system (CNS) results in promptly experienced pleasurable sensations and highly reinforcing effects [13]. When cannabis is smoked, THC concentrations peak slightly lower than those achieved through intravenous administration [13,14]. Dronabinol, usually used for nausea and vomiting in patients undergoing chemotherapy and as an appetite stimulant in human immunodeficiency virus (HIV) wasting disease, may be ingested orally or taken rectally. It is a synthetic form of THC. Oral abuse is also common and leads to lower and delayed peak serum concentration [13,15,16]. Sativex, a form of THC and CBD combined, is administered sublingually, thereby bypassing the liver. It was approved in Canada in 2005 for the treatment of neuropathic pain in patients with multiple sclerosis [13].

As stated above, the primary active compounds in cannabis are delta-9-tetrahydrocannabinol and cannabidiol. THC, the psychoactive component of cannabis, primarily acts on cannabinoid receptor 1 (CB1), found predominantly in the brain and central nervous system. Activation of CB1 receptors leads to various effects, including euphoria, relaxation, altered perception, and appetite stimulation [17]. CBD, on the other hand, interacts with multiple molecular targets, including cannabinoid receptors, serotonin receptors, and transient receptor potential channels. Unlike THC, CBD does not produce intoxicating effects but is associated with potential therapeutic properties, such as analgesia, anti-inflammatory effects, and anxiolytic properties [18]. These facts and statistics have been inferred from the Centers for Disease Control and Prevention (CDC). According to the Substance Abuse and Mental Health Services Administration 2019 survey, marijuana is the most commonly used federally illegal drug in the United States, with 48.2 million people reporting using marijuana at least once in 2019 [19]. Hasin et al. reported that the prevalence of marijuana use doubled in 2012-2013 compared to 2001-2002. Of marijuana users, 30% have a diagnosis of marijuana use disorder according to the Diagnostic and Statistical Manual of Mental Disorders, Fourth Edition (DSM-IV) criteria. Increasing cases of marijuana use disorders are directly linked to increased use of marijuana [20]. Prolonged or regular marijuana usage has been associated with heightened chances of psychosis or schizophrenia development in certain individuals [21]. Utilizing cannabis while pregnant may elevate the likelihood of experiencing pregnancy-related complications. It is advisable for pregnant and breastfeeding individuals to refrain from using marijuana [22].

**Figure 1:**
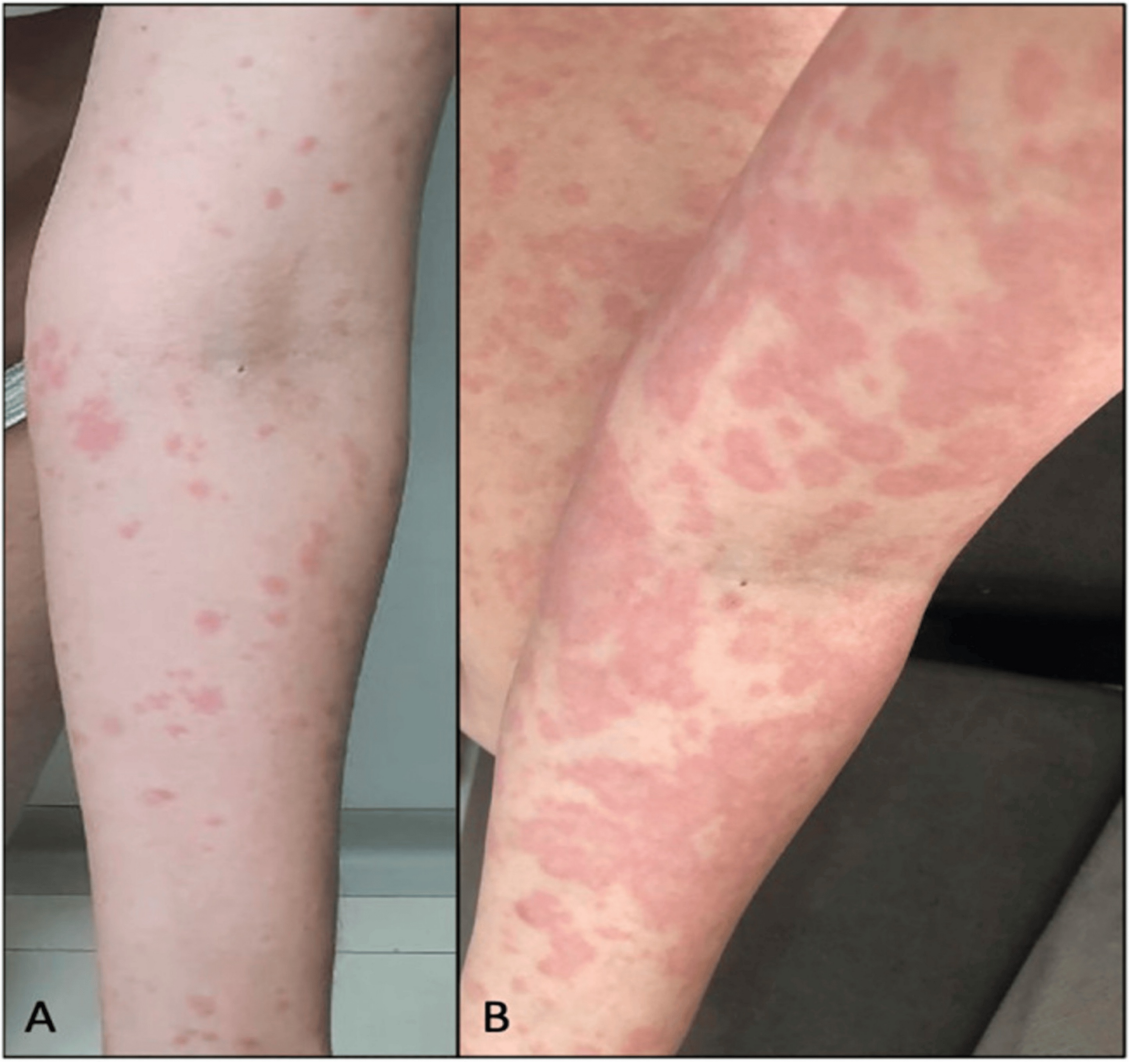
A: Erythematoedematous papules after CBD use. B: Evolution after two days during which CBD was discontinued and oral prednisone was initiated. CBD: cannabidiol Source: Souza JD, Fassoni-Ribeiro M, Batista RM, et al.: Case Report: Cannabidiol-Induced Skin Rash: A Case Series and Key Recommendations. Front Pharmacol. 2022, 13:881617. 10.3389/fphar.2022.881617 [5] Image taken from an open-access article distributed under the terms of the Creative Commons Attribution License. The use, distribution, or reproduction in other forums is permitted, provided the original author(s) and the copyright owner(s) are credited and that the original publication in this journal is cited, in accordance with accepted academic practice. Attribution and permission obtained.

Cannabis hypersensitivity covers a wide range of allergic reactions, including both type 1 and type 4 responses [23,24]. Figure 1 (adapted from Souza et al. [5]) demonstrates erythematoedematous papules after CBD use and evolution after two days during which CBD was discontinued and oral prednisone was initiated.

Cutaneous responses include urticaria, angioedema, generalized pruritus, anaphylaxis, and contact dermatitis. Initial accounts of allergic responses involved cases of contact dermatitis following contact with the cannabis plant, as well as toxicodermatitis subsequent to smoking hemp [23,25]. Obtaining a thorough clinical history is the cornerstone for diagnosing *Cannabis sativa* allergy. Allergenic proteins in cannabis include glyceraldehyde-3-phosphate dehydrogenase, adenosine triphosphate synthase, phosphoglycerate kinase, ribulose-1,5-bisphosphate carboxylase-oxygenase, heat shock protein 70 [23,26], peptinesterases, polygalacturonase [23,27], and thaumatin-like protein [23,28]. Skypala et al. propose a diagnostic algorithm for cannabis allergy. According to them, if history is suggestive of cannabis allergy, then confirmatory testing should be performed using whole extract (WE) [23]. Whole extract tests include skin prick tests, specific IgE testing (using western blot or cytometric bead array), or whole extract basophil activation test (BAT) and whole extract mast cell activation test (MAT). With no readily available extracts for office use, prick tests might be the only alternative for some patients [23,26]. BAT and MAT are ex vivo tests, and they involve analysis and quantification of ex vivo activated basophils or mast cells using recombinant *Cannabis sativa* component (r Can S). If WE tests are negative, cannabis allergy is considered unlikely. If WE tests are positive, molecular testing must be performed to confirm the diagnosis and predict cross-reactivity syndromes. Management strategies include prevention as the mainstay of treatment [23].

Cannabis pollen is an aeroallergen that has been linked to hypersensitivity pneumonitis, allergic rhinitis, allergic keratoconjunctivitis, and worsening of asthma symptoms [29]. An instance of occupational contact urticaria was documented in a forensic sciences worker who spent two years regularly interacting with cannabis at work. She had no history of atopic dermatitis or dermatographism and was not a recreational user either, which suggests that frequent handling was the specific cause of her sensitivity [30]. In another case, an individual with a history of cannabis use presented with a vesiculobullous, scaly, and target-like rash on his extremities that advanced toward his trunk over the course of two weeks and fluctuated in intensity [31].

Darling and Arendorf compared methaqualone, cannabis, and tobacco smokers to non-smokers. The assessment encompassed evaluations of oral tissue integrity and xerostomia. Observed lesions included leukoedema, leukoplakia, and an array of other pathologies. Notably, the only statistically significant disparities in oral manifestations among cannabis users, relative to the control groups, were higher occurrences of leukoedema, dry mouth, and traumatic ulcers [32]. Cannabis users generally experience poorer oral health than non-users, facing a higher risk of dental cavities and gum disease. Cannabis smoke contains carcinogens linked to tissue changes and precancerous lesions in the mouth. Users may be more susceptible to oral infections, likely due to the immunosuppressive effects of cannabis. Dental professionals should recognize these risks and include cannabis use in patient histories to ensure comprehensive care [33]. Cannabis, methaqualone, and tobacco use have been associated with increased *Candida albicans* prevalence and density in oral carriage [34].

Cannabis arteritis is a quite rare peripheral vascular disease with dermatological manifestations and is quite similar to Berger’s disease (thromboangiitis obliterans). El Omri et al. reported one of the few cases of cannabis arteritis in a young female patient who developed digital necrosis in the fingers of her left hand after chronic cannabis and tobacco use. She was treated with prostaglandin, anticoagulation, acetylsalicylic acid, and hyperbaric oxygen. Despite these measures, necrosis did not revert, although it did halt the progression of necrosis. The necrotic sites had to be eventually amputated. Clinical suspicion for cannabis arteritis must remain high in patients with peripheral necrosis and a history of cannabis use. Ultrasound may be used to differentiate from atherosclerosis [35]. Pathogenesis seems to be due to the vasoconstrictive properties of cannabis, as known from animal studies [36]. While the previous paragraphs discussed more well-documented risks of cannabis, there are two initial reports that are worth noting. The first is a report that looked at the structural alterations of the hair shaft with chronic drug use, including cannabis use. Using light and electron microscopy, chronic cannabis users were noted to have local node-shaped enlarged areas that were not seen on the hair shafts of controls [37]. The second is a report of acute generalized exanthematous pustulosis in a 19-year-old woman associated with cannabis use [38]. Obviously, these individual reports require further investigation.

Stevens-Johnson syndrome (SJS) is a rare, serious disorder characterized by a severe, painful rash and blistering of the skin and mucous membranes. It is often triggered by medications or infections and can lead to widespread skin peeling and mucosal ulceration [39]. In 2023, a novel case report described cannabis-induced SJS in a 32-year-old female patient who developed a vesicular and maculopapular rash after using a new cannabis strain. Despite treatment with diphenhydramine, hydrocortisone, and steroids, the rash worsened and spread. SJS was suspected based on painful, small, erythematous, and circular eruptions across her chest and neck. These lesions progressed into edematous blisters that later ruptured. Her psychiatric medications were temporarily stopped, and treatment was adjusted with topical corticosteroids and pain management. Over several days, the rash improved, and she was discharged with instructions to avoid cannabis [40].

A thorough understanding of the risks associated with cannabis consumption is critical for healthcare professionals, particularly those who may encounter patients with a history of cannabis use. Clinicians should know the adverse effects on mucous membranes and the skin, as cessation of cannabis may be necessary.

## Conclusions

Cannabis use, while widespread, carries significant risks, particularly related to dermatological and systemic side effects. Skin reactions, such as rashes and dermatitis, and severe conditions, such as Stevens-Johnson syndrome (SJS), highlight the need for awareness of these risks. Cannabinoids such as THC and CBD can cause a range of skin issues, especially in individuals with allergies or compromised immune systems. Healthcare professionals must be alert to cannabis-induced skin conditions, as prompt diagnosis and treatment are crucial to prevent complications. Additionally, cannabis use has been linked to systemic effects, including cannabis arteritis and oral health problems. With cannabis use increasing, particularly among immunocompromised individuals, the likelihood of severe reactions also rises. Clinicians should be knowledgeable about these potential risks to ensure proper management and patient safety. As cannabis consumption continues to grow, healthcare providers must remain vigilant in recognizing and addressing the dermatological and systemic issues associated with its use. The current review is limited by the scarcity of large-scale dermatology-specific cannabis studies and relies heavily on case reports. Future research should focus on developing standardized diagnostic criteria for cannabis-related skin reactions to improve clinical recognition and reporting. Long-term studies are needed to assess the chronic dermatological effects of cannabis exposure. Clinical trials should evaluate treatment efficacy and safety for these conditions. Additionally, dermatology-specific pharmacovigilance systems must be established to monitor and guide the safe use of cannabinoid-based therapies.
